# Supplementary material for: Large‐scale survey and database of high affinity ligands for peptide recognition modules
Source: Mol Syst Biol. 2020 Dec 8;16(12):e9310. doi: 10.15252/msb.20199310 (PMC7724964; doi:10.15252/msb.20199310)
Supplement: Supplementary file 1 — Appendix [file MSB-16-e9310-s001.pdf]

Table of Contents

Appendix Figure S1: Illustration of specific and non-specific positions in logos.

Appendix Figure S2: Specificity profiles for SH3, WW, and PDZ domains.

Appendix Figure S3: Comparison of phage-derived peptide ligands for PRMs without specificity profiles and structures of peptides in complex with PRMs.

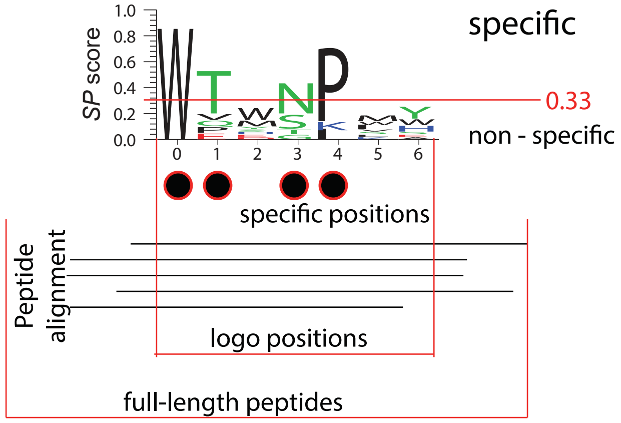

Appendix Figure S1: Illustration of specific and non-specific positions in logos. Specificity profile for a representative PRM (DOK4) with specific and non-specific positions highlighted based on a 0.33 SP score cut-off. A visual depiction of logo and specific positions in the context of an alignment of peptide sequences is shown below the logo.

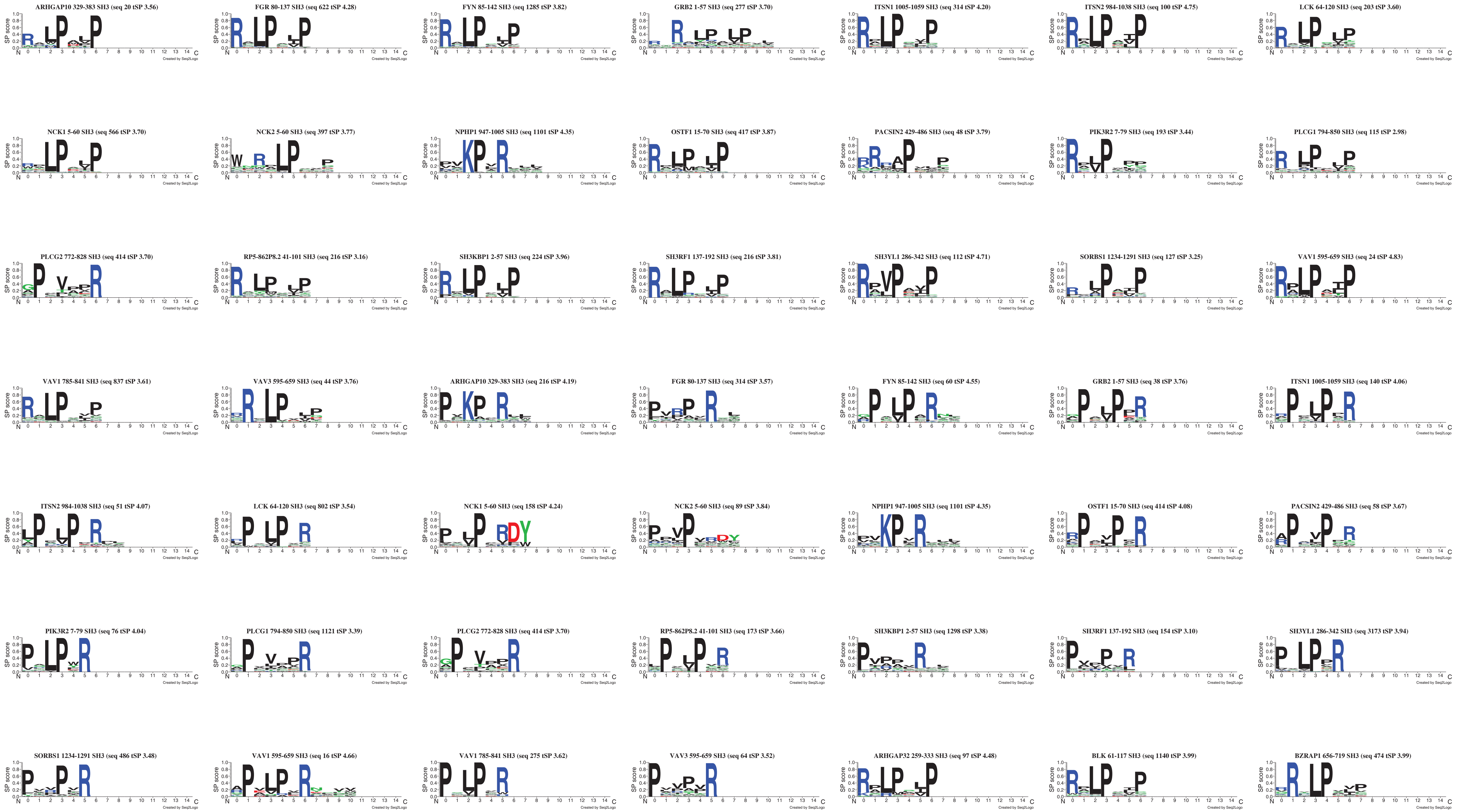
ARHGAP10 329-383 SH3 (seq 20 tSP 3.56)
FGR 80-137 SH3 (seq 622 tSP 4.28)
FYN 85-142 SH3 (seq 1285 tSP 3.82)
GRB2 1-57 SH3 (seq 277 tSP 3.70)
ITSN1 1005-1059 SH3 (seq 314 tSP 4.20)
ITSN2 984-1038 SH3 (seq 100 tSP 4.75)
LCK 64-120 SH3 (seq 203 tSP 3.60)
NCK1 5-60 SH3 (seq 566 tSP 3.70)
NCK2 5-60 SH3 (seq 397 tSP 3.77)
NPHP1 947-1005 SH3 (seq 1101 tSP 4.35)
OSTF1 15-70 SH3 (seq 417 tSP 3.87)
PACSIN2 429-486 SH3 (seq 48 tSP 3.79)
PIK3R2 7-79 SH3 (seq 193 tSP 3.44)
PLCG1 794-850 SH3 (seq 115 tSP 2.98)
PLCG2 772-828 SH3 (seq 414 tSP 3.70)
RP5-862P8.2 41-101 SH3 (seq 216 tSP 3.16)
SH3KBP1 2-57 SH3 (seq 224 tSP 3.96)
SH3RF1 137-192 SH3 (seq 216 tSP 3.81)
SH3YL1 286-342 SH3 (seq 112 tSP 4.71)
SORBS1 1234-1291 SH3 (seq 127 tSP 3.25)
VAV1 595-659 SH3 (seq 24 tSP 4.83)
VAV1 785-841 SH3 (seq 837 tSP 3.61)
VAV3 595-659 SH3 (seq 44 tSP 3.76)
ARHGAP10 329-383 SH3 (seq 216 tSP 4.19)
FGR 80-137 SH3 (seq 314 tSP 3.57)
FYN 85-142 SH3 (seq 60 tSP 4.55)
GRB2 1-57 SH3 (seq 38 tSP 3.76)
ITSN1 1005-1059 SH3 (seq 140 tSP 4.06)
ITSN2 984-1038 SH3 (seq 51 tSP 4.07)
LCK 64-120 SH3 (seq 802 tSP 3.54)
NCK1 5-60 SH3 (seq 158 tSP 4.24)
NCK2 5-60 SH3 (seq 89 tSP 3.84)
NPHP1 947-1005 SH3 (seq 1101 tSP 4.35)
OSTF1 15-70 SH3 (seq 414 tSP 4.08)
PACSIN2 429-486 SH3 (seq 58 tSP 3.67)
PIK3R2 7-79 SH3 (seq 76 tSP 4.04)
PLCG1 794-850 SH3 (seq 1121 tSP 3.39)
PLCG2 772-828 SH3 (seq 414 tSP 3.70)
RP5-862P8.2 41-101 SH3 (seq 173 tSP 3.66)
SH3KBP1 2-57 SH3 (seq 1298 tSP 3.38)
SH3RF1 137-192 SH3 (seq 154 tSP 3.10)
SH3YL1 286-342 SH3 (seq 3173 tSP 3.94)
SORBS1 1234-1291 SH3 (seq 486 tSP 3.48)
VAV1 595-659 SH3 (seq 16 tSP 4.66)
VAV1 785-841 SH3 (seq 275 tSP 3.62)
VAV3 595-659 SH3 (seq 64 tSP 3.52)
ARHGAP32 259-333 SH3 (seq 97 tSP 4.48)
BLK 61-117 SH3 (seq 1140 tSP 3.99)
BZRAP1 656-719 SH3 (seq 474 tSP 3.99)

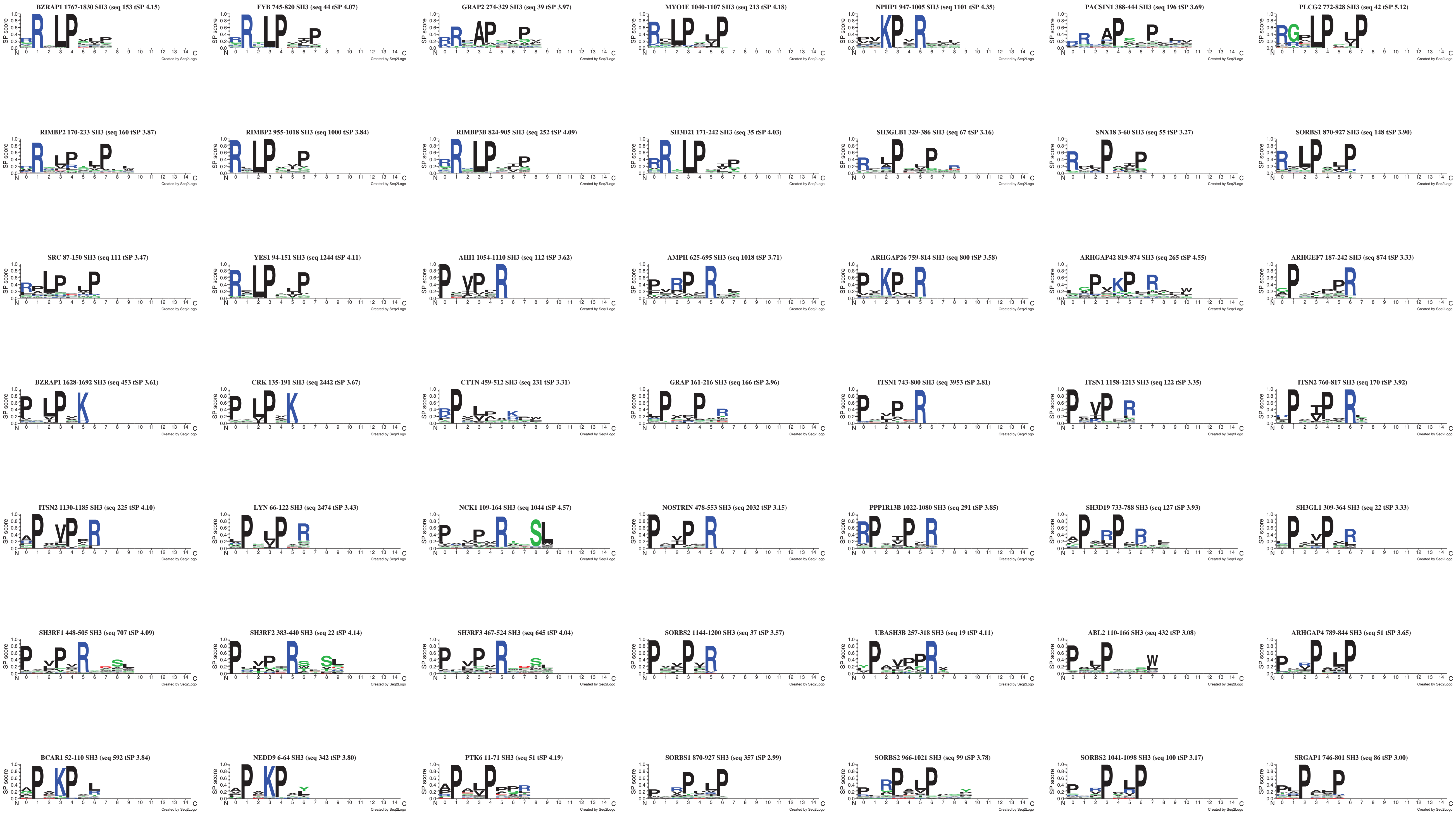
BZRAP1 1767-1830 SH3 (seq 153 tSP 4.15)
FYB 745-820 SH3 (seq 44 tSP 4.07)
GRAP2 274-329 SH3 (seq 39 tSP 3.97)
MYO1E 1040-1107 SH3 (seq 213 tSP 4.18)
NPHP1 947-1005 SH3 (seq 1101 tSP 4.35)
PACSIN1 388-444 SH3 (seq 196 tSP 3.69)
PLCG2 772-828 SH3 (seq 42 tSP 5.12)
RIMBP2 170-233 SH3 (seq 160 tSP 3.87)
RIMBP2 955-1018 SH3 (seq 1000 tSP 3.84)
RIMBP3B 824-905 SH3 (seq 252 tSP 4.09)
SH3D21 171-242 SH3 (seq 35 tSP 4.03)
SH3GLB1 329-386 SH3 (seq 67 tSP 3.16)
SNX18 3-60 SH3 (seq 55 tSP 3.27)
SORBS1 870-927 SH3 (seq 148 tSP 3.90)
SRC 87-150 SH3 (seq 111 tSP 3.47)
YES1 94-151 SH3 (seq 1244 tSP 4.11)
AHI1 1054-1110 SH3 (seq 112 tSP 3.62)
AMPH 625-695 SH3 (seq 1018 tSP 3.71)
ARHGAP26 759-814 SH3 (seq 800 tSP 3.58)
ARHGAP42 819-874 SH3 (seq 265 tSP 4.55)
ARHGEF7 187-242 SH3 (seq 874 tSP 3.33)
BZRAP1 1628-1692 SH3 (seq 453 tSP 3.61)
CRK 135-191 SH3 (seq 2442 tSP 3.67)
CTTN 459-512 SH3 (seq 231 tSP 3.31)
GRAP 161-216 SH3 (seq 166 tSP 2.96)
ITSN1 743-800 SH3 (seq 3953 tSP 2.81)
ITSN1 1158-1213 SH3 (seq 122 tSP 3.35)
ITSN2 760-817 SH3 (seq 170 tSP 3.92)
ITSN2 1130-1185 SH3 (seq 225 tSP 4.10)
LYN 66-122 SH3 (seq 2474 tSP 3.43)
NCK1 109-164 SH3 (seq 1044 tSP 4.57)
NOSTRIN 478-553 SH3 (seq 2032 tSP 3.15)
PPP1R13B 1022-1080 SH3 (seq 291 tSP 3.85)
SH3D19 733-788 SH3 (seq 127 tSP 3.93)
SH3GL1 309-364 SH3 (seq 22 tSP 3.33)
SH3RF1 448-505 SH3 (seq 707 tSP 4.09)
SH3RF2 383-440 SH3 (seq 22 tSP 4.14)
SH3RF3 467-524 SH3 (seq 645 tSP 4.04)
SORBS2 1144-1200 SH3 (seq 37 tSP 3.57)
UBASH3B 257-318 SH3 (seq 19 tSP 4.11)
ABL2 110-166 SH3 (seq 432 tSP 3.08)
ARHGAP4 789-844 SH3 (seq 51 tSP 3.65)
BCAR1 52-110 SH3 (seq 592 tSP 3.84)
NEDD9 6-64 SH3 (seq 342 tSP 3.80)
PTK6 11-71 SH3 (seq 51 tSP 4.19)
SORBS1 870-927 SH3 (seq 357 tSP 2.99)
SORBS2 966-1021 SH3 (seq 99 tSP 3.78)
SORBS2 1041-1098 SH3 (seq 100 tSP 3.17)
SRGAP1 746-801 SH3 (seq 86 tSP 3.00)
SP score
Created by Seq2Logo

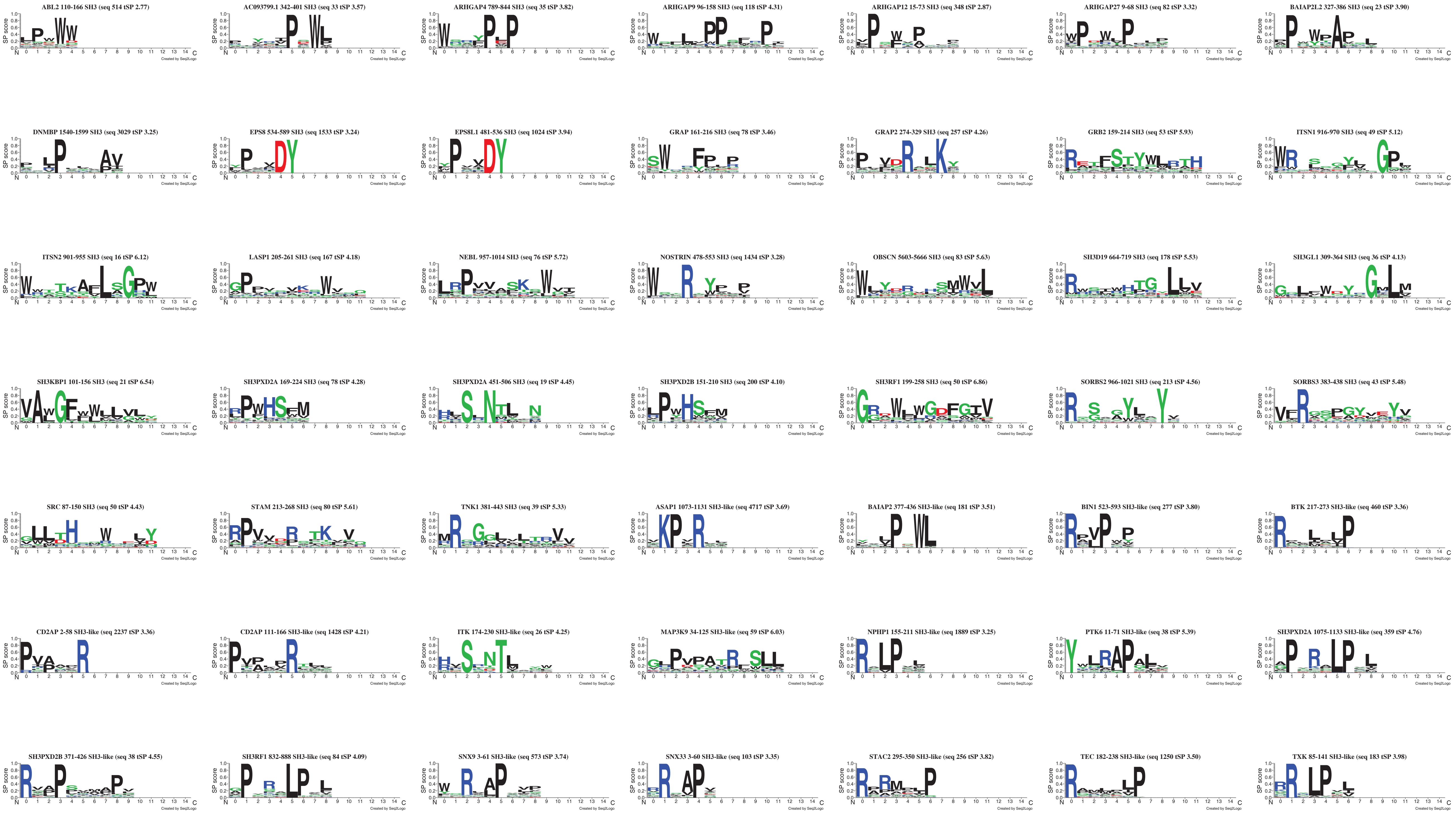
ABL2 110-166 SH3 (seq 514 tSP 2.77)
AC093799.1 342-401 SH3 (seq 33 tSP 3.57)
ARHGAP4 789-844 SH3 (seq 35 tSP 3.82)
ARHGAP9 96-158 SH3 (seq 118 tSP 4.31)
ARHGAP12 15-73 SH3 (seq 348 tSP 2.87)
ARHGAP27 9-68 SH3 (seq 82 tSP 3.32)
BAIAP2L2 327-386 SH3 (seq 23 tSP 3.90)
DNMBP 1540-1599 SH3 (seq 3029 tSP 3.25)
EPS8 534-589 SH3 (seq 1533 tSP 3.24)
EPS8L1 481-536 SH3 (seq 1024 tSP 3.94)
GRAP 161-216 SH3 (seq 78 tSP 3.46)
GRAP2 274-329 SH3 (seq 257 tSP 4.26)
GRB2 159-214 SH3 (seq 53 tSP 5.93)
ITSN1 916-970 SH3 (seq 49 tSP 5.12)
ITSN2 901-955 SH3 (seq 16 tSP 6.12)
LASP1 205-261 SH3 (seq 167 tSP 4.18)
NEBL 957-1014 SH3 (seq 76 tSP 5.72)
NOSTRIN 478-553 SH3 (seq 1434 tSP 3.28)
OBSCN 5603-5666 SH3 (seq 83 tSP 5.63)
SH3D19 664-719 SH3 (seq 178 tSP 5.53)
SH3GL1 309-364 SH3 (seq 36 tSP 4.13)
SH3KBP1 101-156 SH3 (seq 21 tSP 6.54)
SH3PXD2A 169-224 SH3 (seq 78 tSP 4.28)
SH3PXD2A 451-506 SH3 (seq 19 tSP 4.45)
SH3PXD2B 151-210 SH3 (seq 200 tSP 4.10)
SH3RF1 199-258 SH3 (seq 50 tSP 6.86)
SORBS2 966-1021 SH3 (seq 213 tSP 4.56)
SORBS3 383-438 SH3 (seq 43 tSP 5.48)
SRC 87-150 SH3 (seq 50 tSP 4.43)
STAM 213-268 SH3 (seq 80 tSP 5.61)
TNK1 381-443 SH3 (seq 39 tSP 5.33)
ASAP1 1073-1131 SH3-like (seq 4717 tSP 3.69)
BAIAP2 377-436 SH3-like (seq 181 tSP 3.51)
BIN1 523-593 SH3-like (seq 277 tSP 3.80)
BTK 217-273 SH3-like (seq 460 tSP 3.36)
CD2AP 2-58 SH3-like (seq 2237 tSP 3.36)
CD2AP 111-166 SH3-like (seq 1428 tSP 4.21)
ITK 174-230 SH3-like (seq 26 tSP 4.25)
MAP3K9 34-125 SH3-like (seq 59 tSP 6.03)
NPHP1 155-211 SH3-like (seq 1889 tSP 3.25)
PTK6 11-71 SH3-like (seq 38 tSP 5.39)
SH3PXD2A 1075-1133 SH3-like (seq 359 tSP 4.76)
SH3PXD2B 371-426 SH3-like (seq 38 tSP 4.55)
SH3RF1 832-888 SH3-like (seq 84 tSP 4.09)
SNX9 3-61 SH3-like (seq 573 tSP 3.74)
SNX33 3-60 SH3-like (seq 103 tSP 3.35)
STAC2 295-350 SH3-like (seq 256 tSP 3.82)
TEC 182-238 SH3-like (seq 1250 tSP 3.50)
TXK 85-141 SH3-like (seq 183 tSP 3.98)

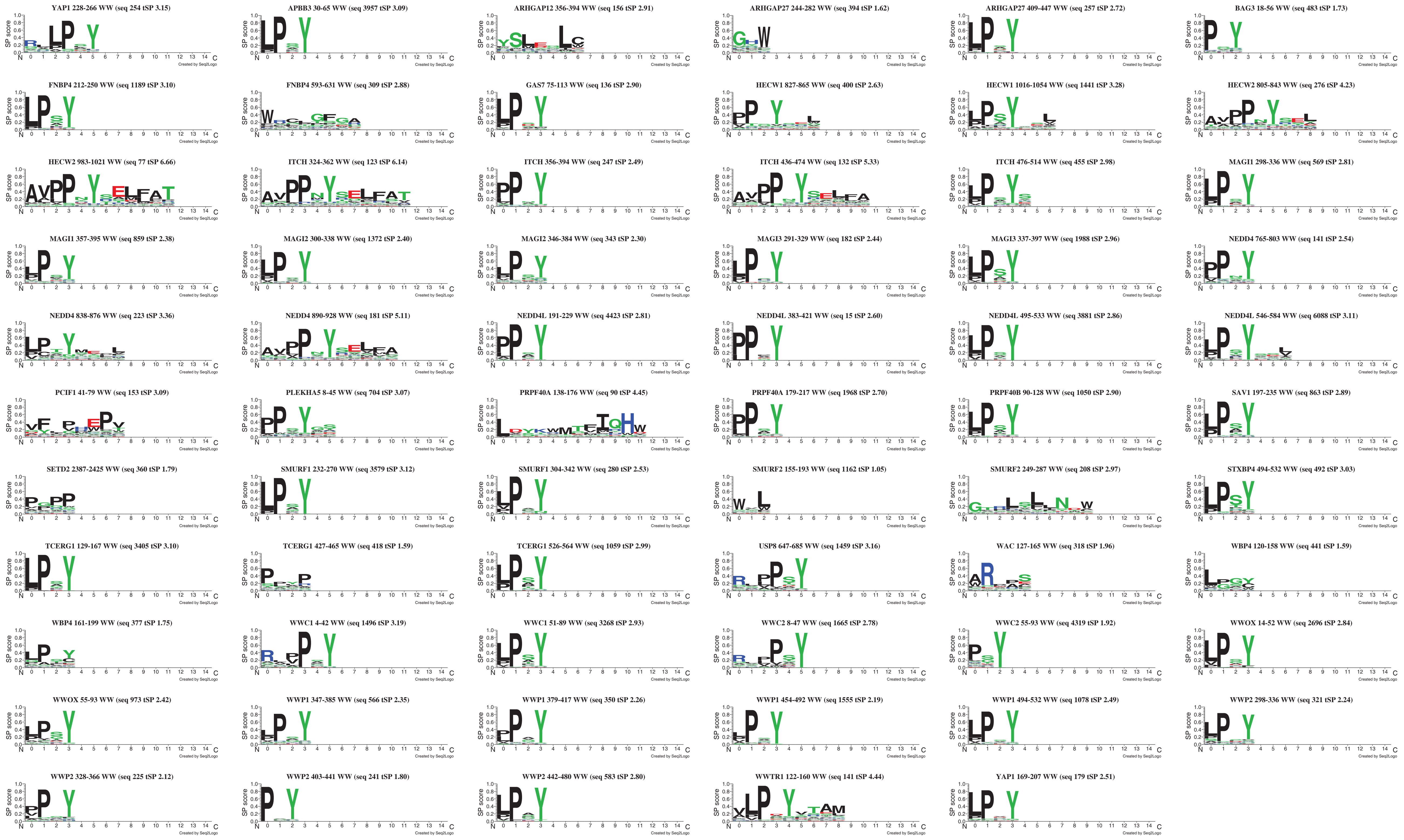
YAP1 228-266 WW (seq 254 tSP 3.15)
APBB3 30-65 WW (seq 3957 tSP 3.09)
ARHGAP12 356-394 WW (seq 156 tSP 2.91)
ARHGAP27 244-282 WW (seq 394 tSP 1.62)
ARHGAP27 409-447 WW (seq 257 tSP 2.72)
BAG3 18-56 WW (seq 483 tSP 1.73)
FNBP4 212-250 WW (seq 1189 tSP 3.10)
FNBP4 593-631 WW (seq 309 tSP 2.88)
GAS7 75-113 WW (seq 136 tSP 2.90)
HECW1 827-865 WW (seq 400 tSP 2.63)
HECW1 1016-1054 WW (seq 1441 tSP 3.28)
HECW2 805-843 WW (seq 276 tSP 4.23)
HECW2 983-1021 WW (seq 77 tSP 6.66)
ITCH 324-362 WW (seq 123 tSP 6.14)
ITCH 356-394 WW (seq 247 tSP 2.49)
ITCH 436-474 WW (seq 132 tSP 5.33)
ITCH 476-514 WW (seq 455 tSP 2.98)
MAGI1 298-336 WW (seq 569 tSP 2.81)
MAGI1 357-395 WW (seq 859 tSP 2.38)
MAGI2 300-338 WW (seq 1372 tSP 2.40)
MAGI2 346-384 WW (seq 343 tSP 2.30)
MAGI3 291-329 WW (seq 182 tSP 2.44)
MAGI3 337-397 WW (seq 1988 tSP 2.96)
NEDD4 765-803 WW (seq 141 tSP 2.54)
NEDD4 838-876 WW (seq 223 tSP 3.36)
NEDD4 890-928 WW (seq 181 tSP 5.11)
NEDD4L 191-229 WW (seq 4423 tSP 2.81)
NEDD4L 383-421 WW (seq 15 tSP 2.60)
NEDD4L 495-533 WW (seq 3881 tSP 2.86)
NEDD4L 546-584 WW (seq 6088 tSP 3.11)
PCIF1 41-79 WW (seq 153 tSP 3.09)
PLEKHA5 8-45 WW (seq 704 tSP 3.07)
PRPF40A 138-176 WW (seq 90 tSP 4.45)
PRPF40A 179-217 WW (seq 1968 tSP 2.70)
PRPF40B 90-128 WW (seq 1050 tSP 2.90)
SAV1 197-235 WW (seq 863 tSP 2.89)
SETD2 2387-2425 WW (seq 360 tSP 1.79)
SMURF1 232-270 WW (seq 3579 tSP 3.12)
SMURF1 304-342 WW (seq 280 tSP 2.53)
SMURF2 155-193 WW (seq 1162 tSP 1.05)
SMURF2 249-287 WW (seq 208 tSP 2.97)
STXBP4 494-532 WW (seq 492 tSP 3.03)
TCERG1 129-167 WW (seq 3405 tSP 3.10)
TCERG1 427-465 WW (seq 418 tSP 1.59)
TCERG1 526-564 WW (seq 1059 tSP 2.99)
USP8 647-685 WW (seq 1459 tSP 3.16)
WAC 127-165 WW (seq 318 tSP 1.96)
WBP4 120-158 WW (seq 441 tSP 1.59)
WBP4 161-199 WW (seq 377 tSP 1.75)
WWC1 4-42 WW (seq 1496 tSP 3.19)
WWC1 51-89 WW (seq 3268 tSP 2.93)
WWC2 8-47 WW (seq 1665 tSP 2.78)
WWC2 55-93 WW (seq 4319 tSP 1.92)
WWOX 14-52 WW (seq 2696 tSP 2.84)
WWOX 55-93 WW (seq 973 tSP 2.42)
WWP1 347-385 WW (seq 566 tSP 2.35)
WWP1 379-417 WW (seq 350 tSP 2.26)
WWP1 454-492 WW (seq 1555 tSP 2.19)
WWP1 494-532 WW (seq 1078 tSP 2.49)
WWP2 298-336 WW (seq 321 tSP 2.24)
WWP2 328-366 WW (seq 225 tSP 2.12)
WWP2 403-441 WW (seq 241 tSP 1.80)
WWP2 442-480 WW (seq 583 tSP 2.80)
WWTR1 122-160 WW (seq 141 tSP 4.44)
YAP1 169-207 WW (seq 179 tSP 2.51)
SP score
Created by Seq2Logo

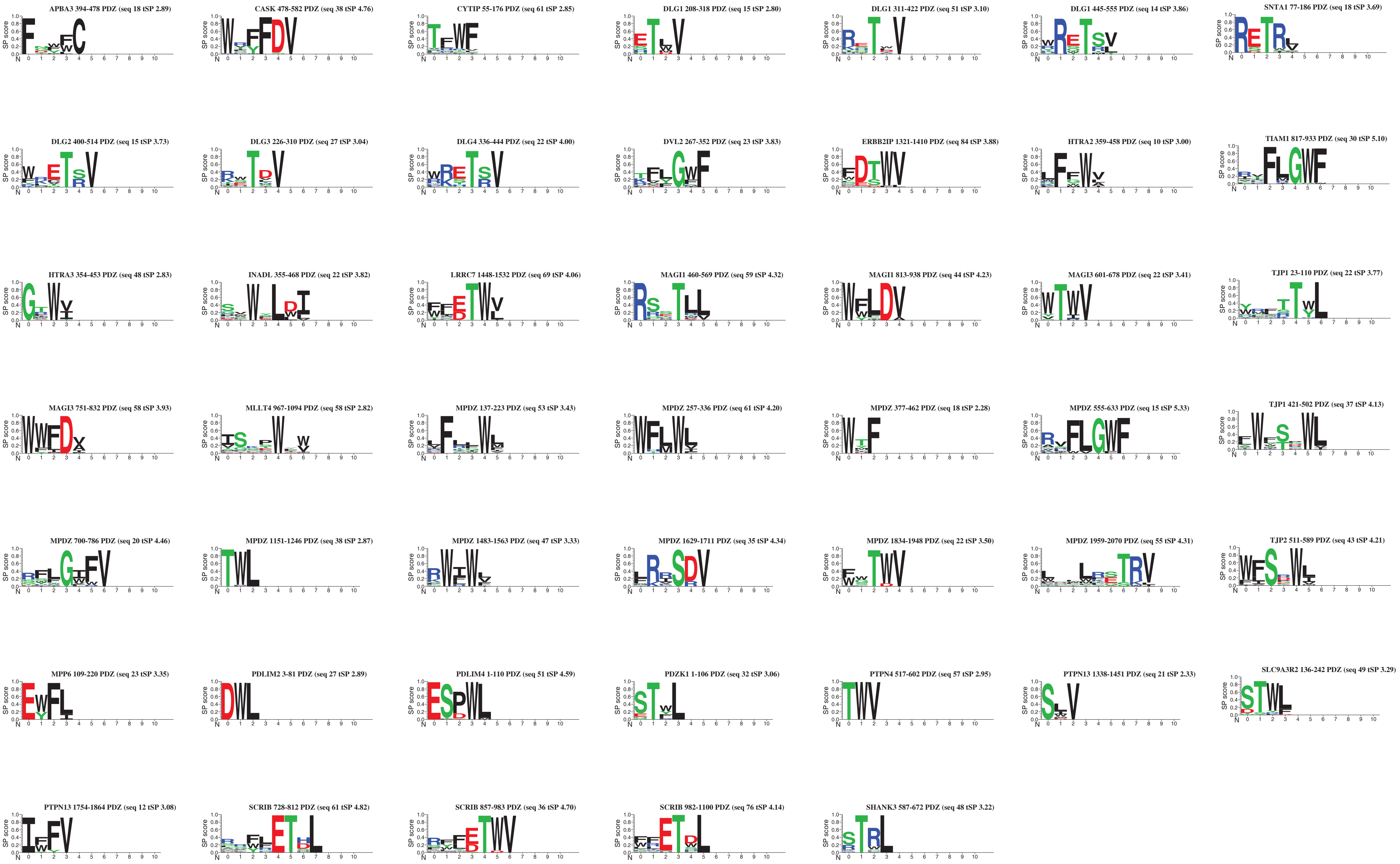

Appendix Figure S2: Specificity profiles for SH3, WW and PDZ domains.
Specificity profiles were calculated using the approach described in the Methods for 115 SH3, 66 WW, and 58 PDZ human domains studied previously (Teyra et al, 2017; Tonikian et al, 2008). The name of the protein is listed at the top with the PRM family name in parenthesis, followed by the specificity profile logo determined from phage-derive peptides.

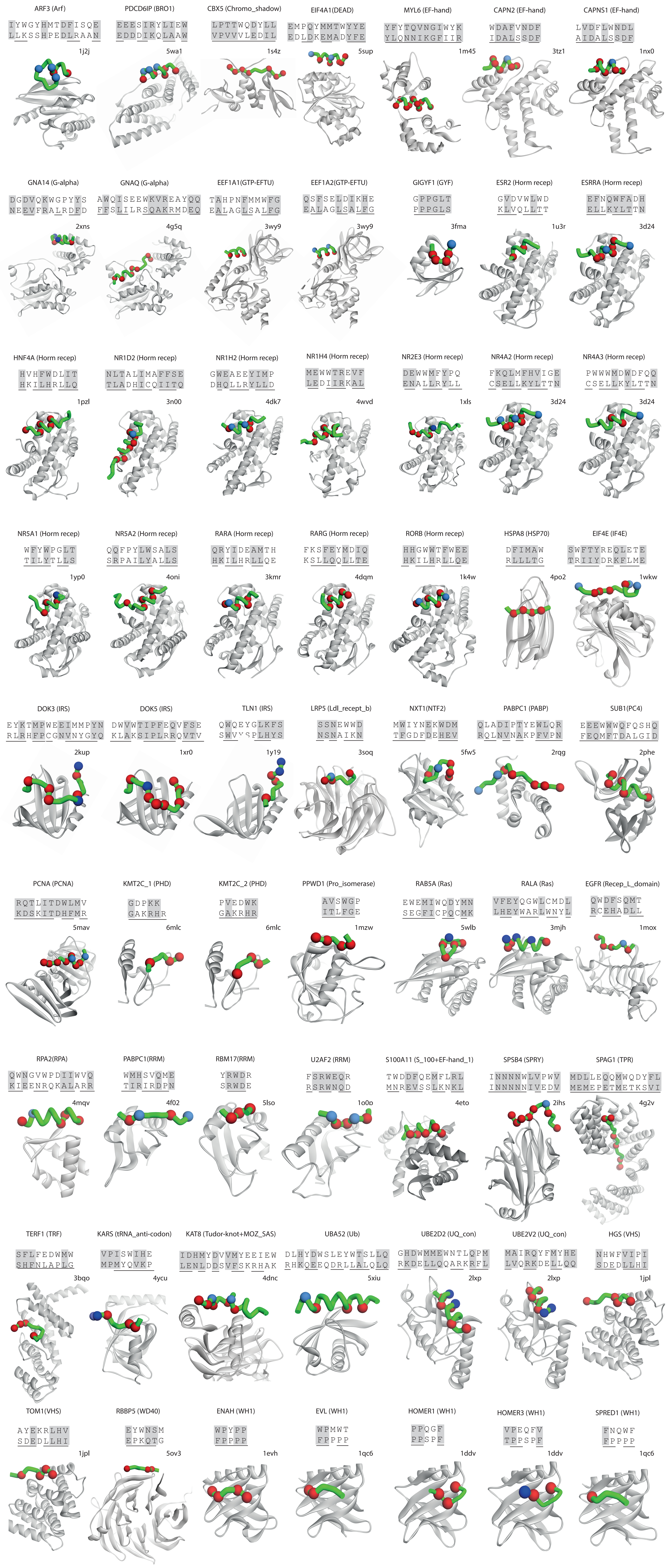

Appendix Figure S3: Comparison of phage-derived peptide ligands for PRMs without specificity profiles and structures of peptides in complex with PRMs. Depicted are the 63 PRMs for which phage-derived peptide ligands were obtained and the structure peptide did not contain a PTM, but for which specificity profiles were not determined. The name of the protein from which the studied PRM was derived is listed at the top with the PRM family name in parenthesis and sorted alphabetically by family. The alignment below the logo shows the sequences of a phage-derived peptide (top) and the peptide ligand from the best-matched PRM-ligand complex structure in the PDB (bottom). Similar residues in the two peptides are shaded gray and residues that make contact with the PRM in the structure are underlined. The structure of the best-matched PRM-ligand complex in the PDB is shown with the PRM and peptide ligand main chains rendered as gray or green ribbons, respectively. Red and blue spheres denote ligand positions that are similar to the phage-derived peptide and are contact or non-contact positions, respectively. The peptide main chain is only depicted for those residues that are shown in the alignment with the phage-derived peptide. The PDB entry code is shown above each structure.
